# Retracted: Ghanaians Might Be at Risk of Inadequate Dietary Intake of Potassium

**DOI:** 10.1155/2017/6195351

**Published:** 2017-07-12

**Authors:** Journal of Nutrition and Metabolism

Journal of Nutrition and Metabolism has retracted the article titled “Ghanaians Might Be at Risk of Inadequate Dietary Intake of Potassium” [1]. The authors have noted that the potassium values should have been multiplied by 10, and this will substantially alter the conclusion of the article.
